# Response to the letter, entitled “Role of hemagglutinin esterase protein in neurological manifestation of COVID-19”

**DOI:** 10.1186/s12987-021-00275-y

**Published:** 2021-09-03

**Authors:** Rashid Deane

**Affiliations:** Department of Neuroscience, University of Rochester, URMC, 601 Elmwood Avenue, Rochester, NY 14642 USA

In the letter to the Editor, entitled “Role of hemagglutinin esterase protein in neurological manifestation of COVID-19”, the authors [1] appear to indicate that a role was attributed to hemagglutinin esterase (HE) in the coronavirus disease 2019 (COVID-19) neurological manifestation in a recent review article [2]. However, in this review HE is mentioned once but there is no discussion on its function. In addition, there is no speculation on HE’s role in COVID-19 and specifically on the neurological manifestations of this infection in the review [2].

At the time the manuscript of the review was prepared, there were articles, which contain diagrammatic figures of severe acute respiratory syndrome coronavirus-2 (SARS-CoV-2) with HE and/or descriptions of HE as a structural component [3–5]. Also, a more recent article described HE as a structural component of SARS-CoV-2, and there is, also, a diagrammatic figure of SARS-CoV-2 with HE [6]. Thus, there is still ongoing confusion on HE’s presence in SARS-CoV-2.

At the time of the COVID-19 outbreak, information on the virus genomic structure, membrane proteins and their functions were rapidly evolving. Indeed, the completion of the genetic sequencing of SARS-CoV-2 led to the production of effective vaccines, and a better understanding of the virus lineage and the origin of its variants. Thus, the information in the literature on whether HE is present in this virus may be due to the evolving knowledge on the virus, including its structure, function of its various membrane proteins and the etiology of COVID-19.

However, the references used to support the statement on the presence of HE in SARS-CoV-2 were inadvertently removed during the editing process of the review [2]. These are now included in this letter [3, 4].

## Data Availability

Not applicable.
